# Gated MoS_2_/SiN nanochannel for tunable ion transport and protein translocation

**DOI:** 10.1186/s12951-025-03991-x

**Published:** 2026-01-09

**Authors:** Shukun Weng, Ali Douaki, Makusu Tsutsui, German Lanzavecchia, Anastasiia Sapunova, Lorenzo Iannetti, Alberto Giacomello, Roman Krahne, Denis Garoli

**Affiliations:** 1 Optoelectronics Research Line, Instituto Italiano di Tecnologia, Genova, 16163 Italy; 2 Department of Materials Science, University of Milano-Bicocca, Via R. Cozzi 55, Milano, I-20125 Italy; 3 Dipartimento di Scienze e Metodi dell’Ingegneria, Universitàdegli Studi di Modena e Reggio Emilia, Via Amendola, 2, Reggio Emilia, 42122 Italy; 4 The Institute of Scientific and Industrial Research, Osaka University, Mihogaoka 8-1, Ibaraki, 567-0047 Osaka Japan; 5 Dipartimento di Ingegneria Meccanica e Aerospaziale, Sapienza Università di Roma, Via Eudossiana, 18, Roma, 00184 Italy

**Keywords:** Solid-state nanopores, 2D material, Nanochannel, Ion transport, Single protein detection, Osmotic power

## Abstract

Ionic transport in nanofluidic channels holds great promise for applications such as single-molecule analysis, molecular manipulation, and energy harvesting. However, achieving precise control over ion transport remains a major challenge. In this work, we introduce a MoS_2_/SiN hybrid nanochannel architecture that enables electrical tuning of ionic transport via external gating, and we examine its potential for osmotic power generation and single-molecule detection. To fabricate the channels, we employed a combined focused ion beam (FIB) milling and dry transfer method, producing sub-10 nm thick structures while preserving the structural integrity and electronic properties of MoS_2_—essential for reliable surface charge modulation. We first investigated how the gate voltage influences ionic conductance, finding evidence of gate-dependent modulation of ion selectivity under different bias polarities, with a rectification ratio of up to 10 in 1 M KCl. Next, by applying a salt concentration gradient across the nanochannels, we demonstrated the feasibility of this platform for osmotic energy generation, achieving a maximum power output of ~ 18 pW from a single channel and ~ 144 pW and ~ 337 pW from 10-channels and 20-channels array, corresponding to a high power density of ~ 18 kW/m². Finally, we tested the system for single-molecule sensing, showing that linearized bovine serum albumin (BSA) produced translocation signals with notably long dwell times exceeding previous reports. Together, these results highlight gated MoS_2_/SiN nanochannels as a promising platform for tunable nanofluidics, with potential applications in controlled molecular transport and energy generation from osmotic gradients.

**Graphical Abstract:**

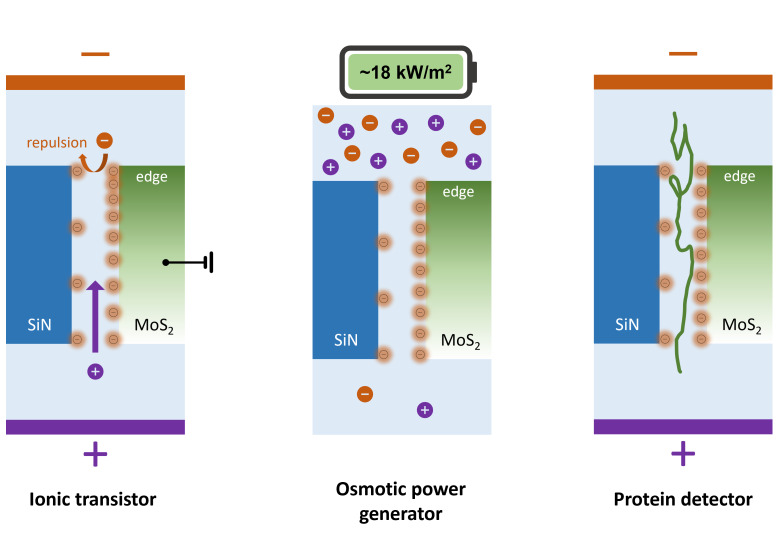

**Supplementary Information:**

The online version contains supplementary material available at 10.1186/s12951-025-03991-x.

## Introduction

Ion transport through nanochannels plays a crucial role in a wide range of applications, including drug delivery, biosensing, desalination, and energy harvesting. Among the various systems studied, nanochannels composed of two-dimensional (2D) materials such as MoS_2_ and graphene have recently received significant attention. Their atomic thickness allows the creation of sub-nanometer channels, which provide a powerful platform for exploring fundamental phenomena such as quantum friction, ion adhesion, orientational conduction, and DNA translocation [1–4]. Despite these advantages, the fabrication of multi-stacked 2D material structures remains a challenging task, and the development of simpler device architectures would greatly facilitate the study of nanofluidic processes in this unique family of devices.

The strong confinement effect characteristic of 2D-material-based nanochannels results in distinctive ion transport properties. These properties can be modulated through adjustments in interlayer spacing, surface chemistry, or material composition [5, 6]. In the context of single-molecule sensing, MoS_2_ offers particular advantages compared with graphene. Its hydrophilic surface reduces unwanted interactions with DNA [4, 7, 8], while intrinsic sulfur vacancies on the MoS_2_ surface provide sites that can selectively slow down or capture proteins containing free thiol groups. This capability highlights MoS_2_’s potential in advanced protein sensing applications [9, 10]. Among emerging nanofluidic device concepts, the ionic transistor stands out as a promising building block for ion-based logic computing. These devices operate by electrically modulating the surface charge of nanopores or nanochannels to control ion transport [11–21]. Two-dimensional materials such as MoS_2_ are especially promising for this purpose, as their carrier type can be efficiently tuned through chemical modification or electrostatic gating [22–27]. However, most existing designs rely on symmetric, suspended 2D membranes that are mechanically fragile and require complex fabrication, limiting reproducibility and scalability.

To address these limitations, we developed a hybrid nanochannel platform that integrates a mechanically robust MoS_2_ flake with a freestanding silicon nitride (SiN) membrane. Unlike conventional 2D material stacks [28–30], the device is fabricated by transferring a thick MoS_2_ flake onto a FIB-milled trench in SiN, forming an asymmetric channel supported on one side by SiN and electronically active on the other. This architecture provides mechanical stability while preserving the tunable electronic properties of MoS_2_ and, crucially, enables selective electrical gating of only the MoS_2_ wall. This selective gating produces a programmable charge asymmetry within the channel, an effect that is not accessible in symmetric 2D nanopores. As a result, the platform allows controlled modulation of ion transport through spatially localized gating rather than relying on geometric asymmetry or chemical patterning.

In this work, we present a comprehensive study of ion transport in electrically gated MoS_2_/SiN nanochannels. We systematically examined how gate voltage, ion concentration, and pH influence surface charge and ion conductance, and we demonstrate the platform’s functionality in two applied contexts: osmotic energy generation and single-protein translocation sensing. Our results reveal gate-dependent rectification behavior, competitive osmotic power densities, and prolonged protein dwell times, highlighting the versatility of this hybrid platform for both energy-harvesting and biosensing applications. Through these experiments, we elucidate possible mechanisms of ion translocation in MoS_2_/SiN nanochannels and discuss their implications for the design of nanofluidic devices. Overall, our findings contribute to the expanding knowledge of nanoscale ion transport and highlight the promise of electrically tunable MoS_2_/SiN nanochannels as a platform for future advances in nanotechnology and biomedicine.

## Experimental methods

### SiN membrane fabrication

Freestanding Si_3_N_4_ membrane chips were fabricated using a standard procedure. An array of square membranes was created on a commercial double-sided 100 nm LPCVD SiN-coated 500 μm Si wafer through UV photolithography, reactive ion etching, and KOH wet etching [31]. Depending on the dimension of the photolithography mask, we can get different sizes of SiN membrane. Here we mainly used a ~ 50*50 µm membrane to increase the robustness of our device and reduce the noise level [32].

### Nanostructure fabrication

**Fig. 1:**
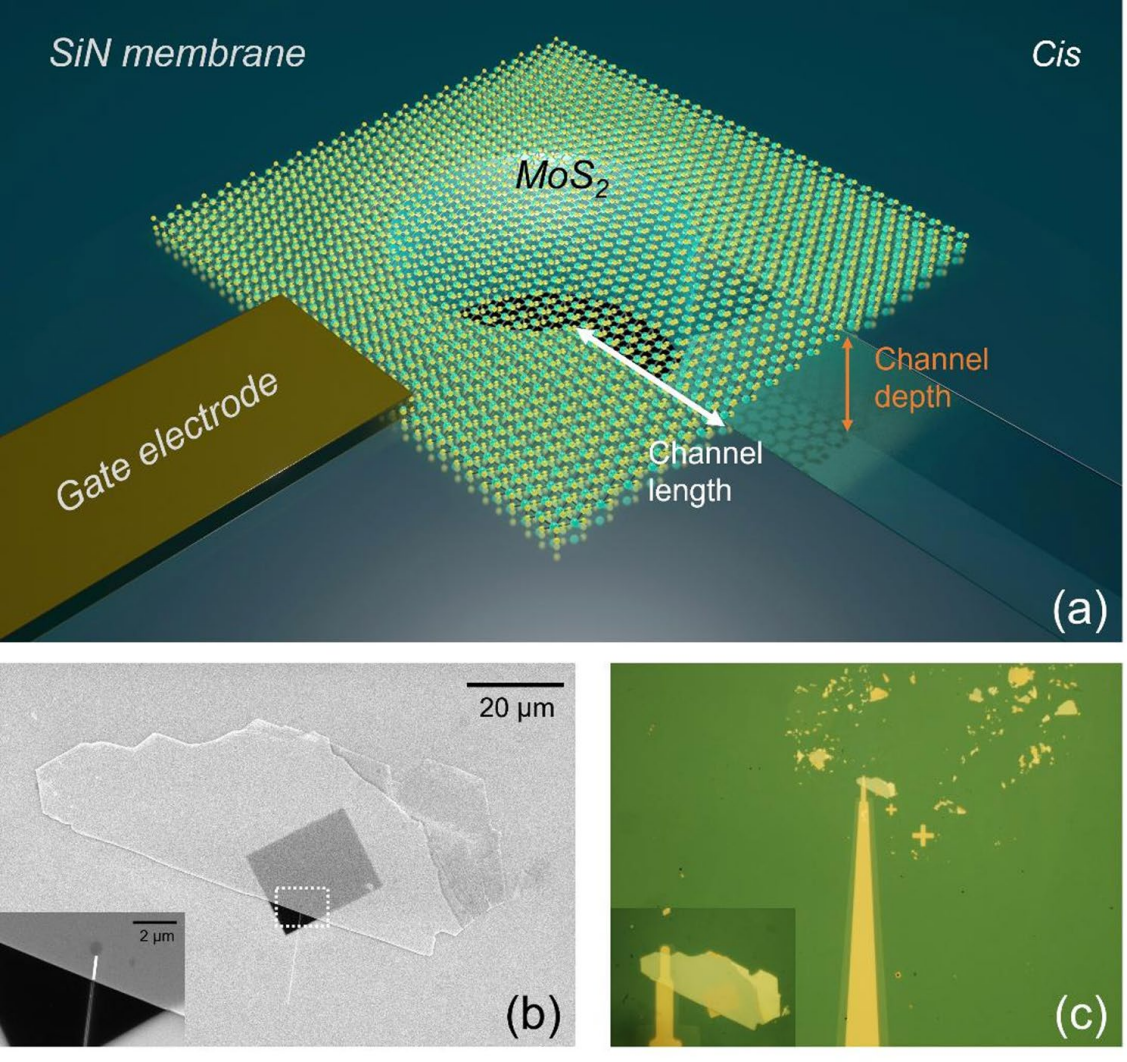
Device design. (**a**) Illustration of nanochannel devices formed by MoS_2_ flake and SiN membrane with a nanopore and nano-slit. The length, width, and height of the nanochannel is around 1 μm, 100 nm, and 10 nm respectively; (**b**) SEM image of nanochannel device just after the transfer of MoS_2_; the inset shows the magnification image of white dash line framed region, and the solid white line in the inset image; (**c**) Optical image of nanochannel device after the Au electrode deposition

Here, we propose a novel structure which can be fabricated by a simpler process compared with previously reported sandwiched 2D material stacks structures [28]. The nanopore and the nano-slit(s) are directly milled by focused ion beam (FIB) on suspended SiN membrane with 30 kV acceleration voltage and 18 pA current, after which a thick layer of MoS_2_ is transferred on top of the nano-slit to form a nanochannel comprised by SiN and MoS_2_ surface, as shown in Fig. 1a. The MoS_2_ flake is typically made thicker than 50 nm to prevent sagging that could obstruct the channel, while still preserving surface charge tunability, since electrons primarily accumulate near the surface. The nanochannel depth is primarily determined by the ion-beam dwell time during milling. In our case, it typically ranges from 5 to 20 nm, as confirmed by AFM measurements on multichannel samples. (Fig. S1).

### 2D material transfer

There are mainly two ways to prepare MoS_2_ flakes, physically by means of mechanical exfoliation (cleave the original crystal) or chemically (controlled chemical reaction on substrate by CVD/CVT). These two techniques result in different surface charge characteristics [33–35]. On the fresh cleaved MoS_2_ flakes, the surface is near intrinsic because of the smooth surface, and the electrons will gradually accumulate on the surface because of the slow desulfurization in the air, which induces a negatively charged MoS_2_ surface.

On the other hand, the CVD/CVT grown MoS_2_ flakes are always negatively charged because of the dangling bonds generated during the chemical reaction process [35]. MoS_2_ flakes prepared via chemical processes can be suspended in proper solvents and easily deposited on solid-state substrates [36] but they typically present a high level of contamination making them not suitable for the scope of this work. Therefore, here, we used exfoliated MoS_2_ flakes as a top layer to form the nanochannels (Fig. 1b). The MoS_2_ flakes were exfoliated using well established scotch tape methods onto a 300 nm SiO_2_ substrate. By using a standard optical microscope, it is possible to quickly obtain a first check on the quality of the transferred flakes. A polycarbonate (PC) thin film on top of PDMS dome supported by a transparent cover slide is used to transfer the selected MoS_2_ flake from SiO_2_ substrate to SiN membrane which has a nanopore connect with a shallow nano-slit (Fig. 1a) [37, 38]. The PC thin film was prepared by a dip-coating method. First, a PC solution was prepared by dissolving polycarbonate in chloroform (10% weight ratio). The solution was then deposited onto a clean glass slide using a drop-casting method. A second glass slide was immediately positioned parallel above the coated slide and gently pressed with finger pressure to eliminate air bubbles between the two surfaces. Subsequently, the upper slide was rapidly removed through a horizontal sliding motion, leaving a thin, uniform layer of PC solution on the lower substrate. The chloroform solvent was allowed to evaporate under ambient conditions for 1 min, resulting in the formation of an ultrathin, uniform PC film. This free-standing PC film, when combined with perforated double-sided adhesive tape mounted on a PDMS stamp, served as an effective transfer medium for 2D materials in subsequent processing steps [39].

### Gating electrode fabrication

The Ti/Au electrode (Fig. 1) was fabricated using two photolithography steps. First, the Au electrode region pattern was defined using the resists LOR3B/S1805 spin coated as a double layer. A 5 nm Ti adhesion layer followed by a 30 nm Au layer was deposited on developed regions by using electron beam evaporation, followed by lift-off in PG remover for 5 min.

A second lithography process using a larger line-width pattern to isolate the Au electrode was performed using a similar process (spin coating photoresist, mask-aligner exposure, evaporation of isolated material and lift-off). As shown in Fig. 1c, the tip of the Au electrode contacts the MoS_2_ flake, while the pad is positioned outside the membrane for electrical characterization of ionic transistor behavior.

### Electrochemical measurements

All electrochemical measurements, except for gating, were carried out using a Nanopore Reader (100 kHz) and PMMA flow cells from Elements srl. Prior to each measurement, the nanochannel device was immersed in an ethanol–water (1:1) solution for 10 min to ensure proper wetting. Two PDMS blocks with central openings were placed on both sides of the device to facilitate mounting in the cell without leakage. After introducing 70 µL of electrolyte into each reservoir, a constant voltage was applied for 10 s in 100 mV (or, when required, 20 mV) increments from V– to V+. For osmotic power measurements, triangular voltage sweeps from − 500 mV to 500 mV were performed at 10 mV/s to determine the intercept value.

For gating experiments, a Keithley 2612B connected to a probe station was used to apply the three-terminal configuration shown in Fig. S2. In this setup, the source–drain potential was swept from − 500 mV to 500 mV over 100 s (10 mV/s).

## Results & discussion

### Basic ion transport properties

**Fig. 2:**
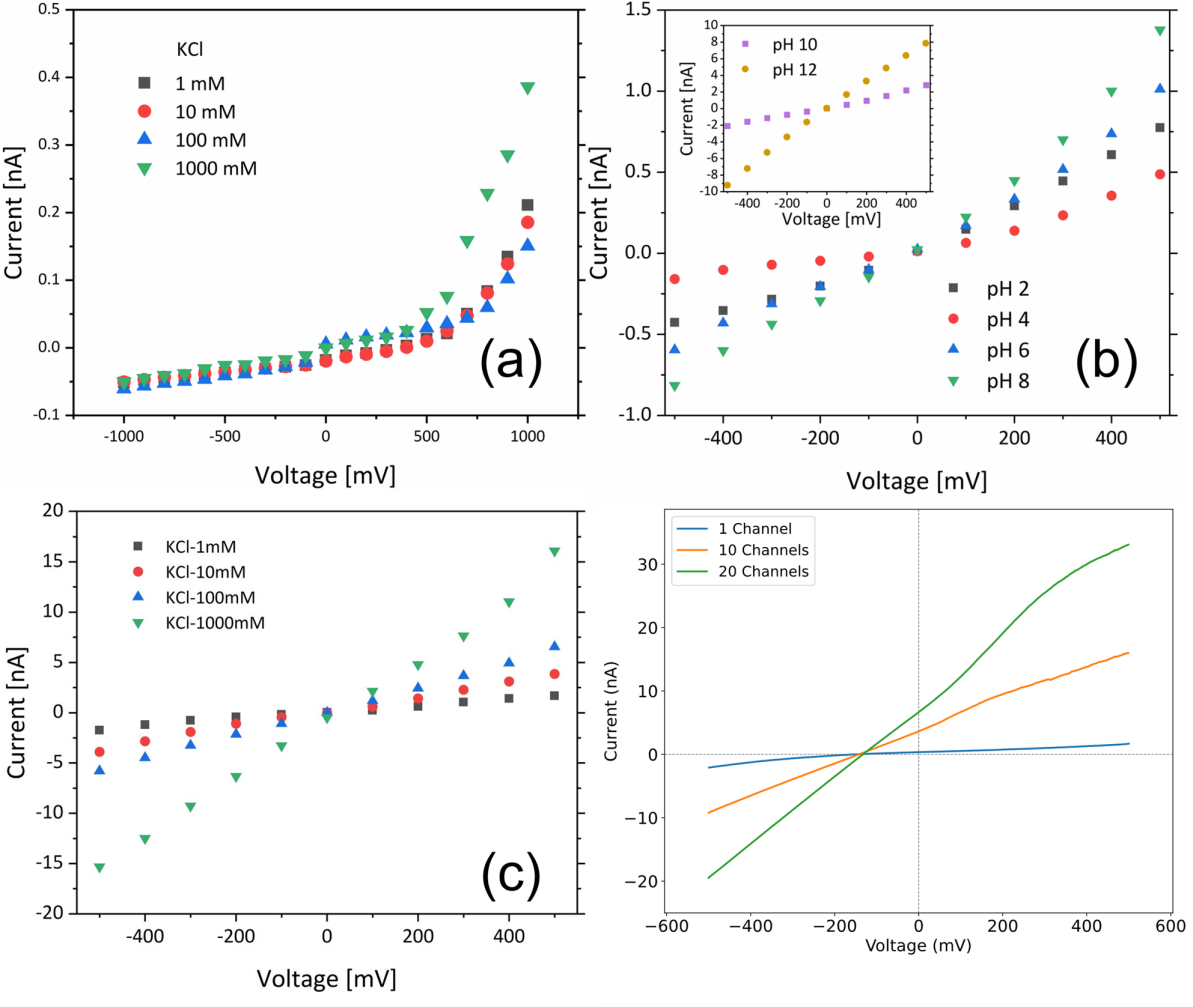
Ion transport of nanochannel. (**a**) Ion current across nanochannel with different KCl concentrations under pH 7.4 before cleaning process and (**b**) Ion current of 10 mM KCl across nanochannel with different pH after cleaning process; (**c**) Ion current of single nanochannel device with different KCl concentration after the cleaning process; (**d**) Nanochannel Ion current with different number of channels under 1X:10X PBS gradient

The first characterization of the platform was done by measuring the ionic current with different concentrations of KCl. The conductance of the nanochannel did not increase linearly when the concentration of KCl electrolyte increased from 1 mM to 1000 mM. This non-linear behavior could be attributed to mechanisms such as ionic coulomb blockade as described for sub-nm monolayer MoS_2_ nanopore or the predominance of counterions transport [40, 41]. While the ion current is very small due to the limited channel height and because of potential hydrocarbon contamination, we observed an ionic rectification with a rectification ratio up to 10 for 1 M KCl as shown in Fig. S3. In order to verify these first results, we repeated the measurements after a cleaning procedure on the sample. The cleaning process was conducted by applying a potential ranging from − 100 mV to 100 mV with an alternating current scan overnight. The alternating electric field prompted ions to accumulate at the nanochannel entrance and repeatedly penetrate the contaminant barrier. Through multiple cycles, the blockages were gradually removed, thereby restoring the ion transport capacity of the channel (Fig. S4) [42]. After the cleaning procedure, a larger and more linear ion current was detected (Fig. 2c). It indicates more symmetric surface charge distribution after the electrolyte cleaning process, as well as the minimal geometric asymmetry.

In order to study the change of surface charge density of the nanochannel system towards ion current flow, we measured the ion current with a different pH of 10 mM KCl. Figure 2(b) shows how the ion current conductance is significantly affected by the pH value of the electrolyte after the cleaning process.

As the pH increases, the current increases, suggesting that the nanochannel surface becomes more negatively charged, attracting the positively charged ions. The minimal ion current value occurred at pH 4 attributable to the effective isoelectric point of the MoS_2_/SiN channel, consistent with published isoelectric point data of MoS_2_ and SiN [43, 44]. With a pH increased from 10 to 12, the rectification direction changed. This phenomenon can be attributed to the distinct deprotonation behaviors of the two channel walls. While both MoS_2_ and SiN remain negatively charged in this alkaline range, their surface charge densities evolve differently: SiN, with an isoelectric point near pH 5–6, is already strongly deprotonated at pH 10, whereas MoS_2_ continues to gain substantial negative charge between pH 10 and pH 12. This shift in the relative surface charge asymmetry between the MoS_2_ and SiN walls alters the spatial distribution of ion enrichment and depletion zones under bias, thereby inverting the rectification direction without requiring a reversal of absolute charge polarity. The pH-dependent behavior highlights the importance of surface charge in controlling ion transport through the nanochannel [45]. The surface charge density of the MoS_2_/SiN nanochannel system can be estimated by ionic conductance measurements, as used for hBN nanotube system [46]:

1 $$\:G=2{e}^{2}\mu\:{C}_{s}\frac{A}{L}+e\mu\:\frac{C}{L}\left|\sigma\:\right|\left(1+\alpha\:\right)$$

Where *e* is the electron charge, *C*_*s*_ is the ion number density of the electrolyte, *A* and *C* are, respectively, the area and perimeter of the nanochannel cross-section, *L* is the length of the channel, *µ* is the mean ion mobility of the electrolyte in s/Kg, *s* is the surface charge density, and *a* which account for the electro-osmotic conductance contribution (here his value is around 1).

As shown in Fig. S5, the surface charge density of our MoS_2_/SiN nanochannel system is around 100 mC/m^2^, much higher than the measured surface charge density of SiN nanopore (~ 15 mC/m^2^) [44], indicating that the MoS_2_ surface plays a more important role in controlling the ion current of our hybrid nanochannel system.

The high surface charge value further shed light on energy harvesting from salinity gradients. Several recent studies, in fact, demonstrated how it is possible to generate energy from the osmotic process in nanopores/nanochannels using the buffers at the cis/trans sides with two significantly different concentrations [11, 47–52]. Here, we first measured the response of our single nanochannel platform using 10X PBS buffer solution (1.37 M NaCl) in both side reservoirs (Cis/Trans) obtaining almost zero ion current without applying any external voltage. On the contrary, replacing the electrolyte in the trans reservoir to 1X PBS (0.137 M NaCl) we immediately got a positive ion current. By scanning the external potential, we can get IV curves from different configurations as shown in Fig. 2(d). From these data we can calculate the power of our osmotic generator to be ~ 18 pW (calculated as $\:P={{V}_{osm}}^{2}G$, where $\:{V}_{osm}={V}_{intecept}-{V}_{redox}$ is the effective osmotic potential and G is the conductance [53]). The redox voltage, $\:{V}_{redox}$ is here set to −61.7 mV, as measured from the open voltage of 1000 nm diameter SiN nanopore under the same saline condition (see Supporting Information - Fig. S6) [49]. The power density of our nanochannel can also be derived by considering the power per unit channel surface of the cross section $\:{P}_{density}={P}_{osm}/A$, where the area of the cross section A is 1000 nm^2^. Using these values the power density of the single nanochannel was calculated to be 18 kW/m^2^, a value higher than that of hBN nanotubes (~ 3 kW/m^2^) [46] and polyimide nanochannel (~ 3.5 kW/m^2^) (see Table S1). To explore a potential scale-up of this system, we have prepared a few more samples with 10 or 20 nanochannels distributed around a single MoS_2_ window as depicted in Fig. S7. The results show sub-linear short-circuit current (I_short_) enhancement while the open-circuit voltage (V_open_) remains at a similar level. We can obtain the average osmotic power density of 10 channels device and 20 channels device as 14,425 W/m^2^ and 33,723 W/m^2^, respectively. The power densities reported here for both single and multi-channel devices are normalized to the total cross-sectional area of the active nanochannel(s). This approach is commonly used in fundamental nanofluidic studies to evaluate the intrinsic efficiency of channel material and structure. The consistent power density across devices with different numbers of channels demonstrates the robustness and scalability of our platform. All multi-channel devices are fabricated in the same plane, with minimal ion concentration polarization (ICP) due to the long channel length and small gradient difference.

### Electrically tunable ion transport

**Fig. 3:**
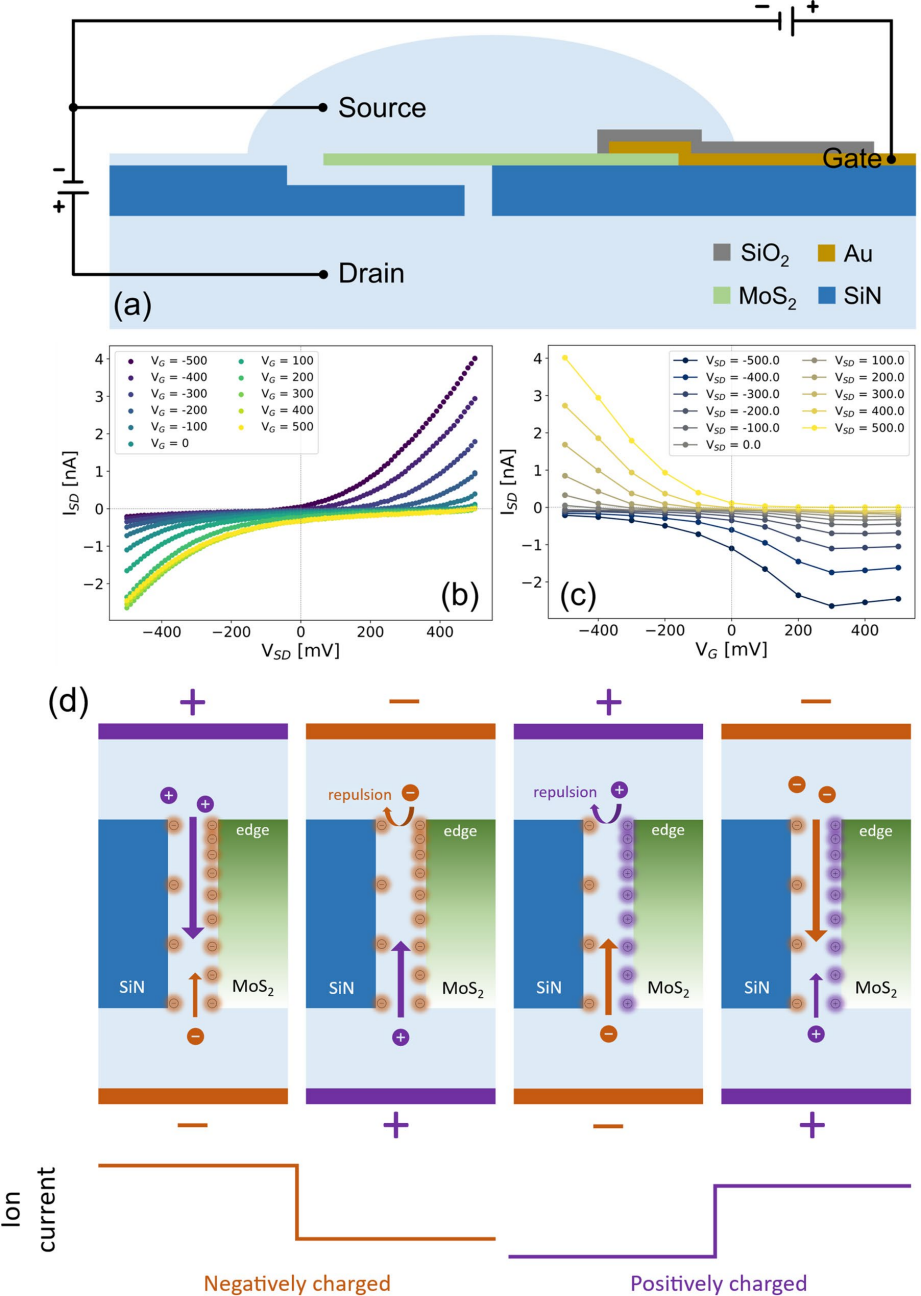
Electrically tunable ion transport of MoS_2_/SiN nanochannel. (**a**) Illustration of measurement configuration; (**b**,**c**) Ion current of nanochannel under different gate bias and transmembrane voltage “mV” in 10 mM KCl. (**d**) Illustration of ion rectification mechanism for the nanochannel

Given that the ion current rectification (ICR) observed in the MoS_2_/SiN nanochannel may result from an unbalanced surface charge distribution between the two channel walls, we probed ion transport as a function of the MoS_2_ surface charge by applying a gate potential through a top electrode. Figure 3(a) shows the electrical measurement setup for the transistor-like configuration, where we used two channels from a Keithley 2612B source meter. As shown in Fig. 3(b), the channel conductance increases with decreasing gate voltage down to −500 mV, which can be attributed to an elevated counter-cation concentration driven by the enhanced negative surface charge on the nanochannel walls. As mentioned in the discussion of Fig. 1, the surface charge of MoS_2_ accounts for a larger proportion of the hybrid system, and the charge of the MoS_2_ is mainly constrained on the surface [54]. The negative surface charge density enhancement under negative gating bias has been shown in many previous KPFM study papers [55–57]. The surface charge reversal has also been predicted via Density Functional Theory (DFT) study by Mortier et al. [58] which could be the mechanism that lies down under our observation, where with applied positive gate voltage we observed a conductance enhancement in the other direction.

These ambipolar characteristics indicate that the nanochannel surface is nearly electrically neutral at zero gate voltage, and the application of either positive or negative gate voltages increases the accumulation of counter-ions, thereby increasing conductance which has also been proved in CNT base ionic transistor system [13, 59].

The I–V curves further reveal a switch in ICR behavior depending on the direction of the applied bias. At zero gate voltage, the current is suppressed under positive transmembrane bias, a trend that persists with increasing positive gate voltage. However, when negative gate voltage is applied, the rectification behavior reverses — conductance under negative transmembrane bias decreases relative to that under positive bias. Considering the indistinctive ion rectification observed in our nanochannel system without an applied gate bias (particularly after voltage-scan cleaning process), the channel geometry and the surface charge distribution can be considered symmetric. The pronounced ion rectification emerges clearly after applying an external gate bias to the MoS_2_ flake, which we attribute to the accumulation of surface charge near the channel edge under small gating bias, an effect consistent with prior microwave impedance microscopy (MIM) characterization on MoS_2_ transistor [60]. As illustrated in Fig. 3d, under a negative gate bias the surface of the MoS_2_ becomes more negatively charged, facilitating cations transport enter from the MoS_2_ edge side of the channel while impeding anion passage if the transmembrane potential is oriented inversely. The opposite side of the channel exhibits lower ion-selectivity due to its weaker surface charge density. With positive charge accumulation, the anions are favored from MoS_2_ edge side while the cations are repelled, creating a bias-dependent asymmetry in ionic current. Furthermore, the non-zero ion current recorded at V_sd_= 0 V under gate bias can be explained by a gate induced built-in potential across the membrane, arising from modulated surface charge asymmetry, which drives ions through the nanochannel in the absence of transmembrane voltage. Similar phenomena have been reported in previous study on ZnO/SiN bilayer nanopores [49] and CNT ionic transistor [13]. Figure 3(c) displayed the gate-dependent ionic current characteristics of our ionic transistor, where an asymmetry can be observed between the response under positive and negative transmembrane potential *V*_*SD*_. For negative *V*_*SD*_, the ion current quickly saturates at around 300 mV while for positive *V*_*SD*_ the *I*_*SD*_ increases monotonically. This asymmetric behavior could be attributed to the asymmetric of the MoS_2_ surface area on cis or trans reservoir, while the surface charge of the smaller area reaches the limited surface charge density faster.

### Protein translocation

In a previous study, Dekker’s group showed that the 2D material stacks nano-slit (nanochannel) can detect different types of DNA translocation events, thereby introducing such nanochannels as a new tool to probe biopolymer properties [4]. Here we tested the ability of our nanochannel for protein translocations measuring the translocation of BSA. Unlike the evenly distributed negative charge and long strand structure of DNA molecules, protein molecules are normally globular which means the dwell time is quite small which makes detection challenging. The unevenly charge distribution makes it difficult for the electrical force driving the protein molecule translocating through the nano-pores/channels [61]. Sodium dodecyl sulfate (SDS) has been proved to unfold protein and stabilize this denatured state. The absorbed SDS alongside the unfolded protein chain can also introduce uniformed negative charge which can facilitate its electrophoretic translocation through 10 nm solid-state nanopore [62, 63].

**Fig. 4:**
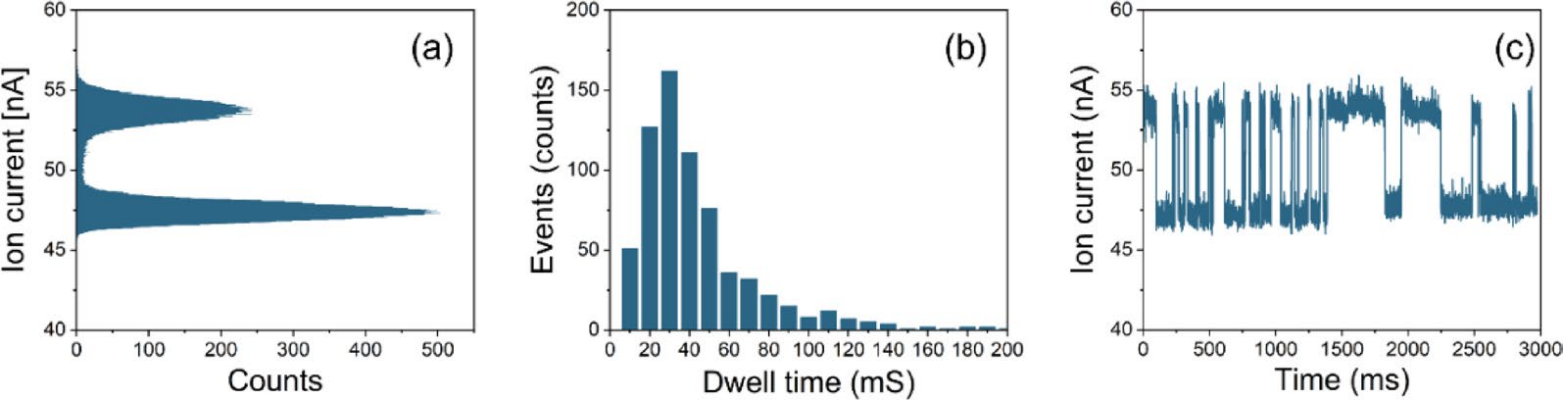
Translocation analysis of BSA through the MoS2/SiN nanochannel under an applied voltage of + 200 mV. (**a**) Ion current blockade histogram; (**b**) Dwell time distribution and (**c**) Section of translocation trace

According to the literature, measuring the magnitude of translocation spikes of BSA protein molecules through ~ 55 nm SiN nanopore with 20 nm thickness, it is possible to estimate the size of the BSA between 7 and 9 nm [64]. After unfolding SDS, the hydrodynamic radii of BSA-SDS were determined to be around 5.9 nm [65]. Here, we used 1 M KCl and 200 µM SDS solution buffered with Tris-EDTA at pH 8 as the electrolyte to measure the translocation of BSA (~ 8 mg in 4 mL TE buffer solution, ~ 30 µM). In our MoS_2_/SiN nanochannel, a voltage of 200 mV is sufficient to initiate the translocation of BSA-SDS chains, which is sufficiently low to prevent damage or delamination of the MoS_2_ layers [28]. An ionic current trace of the MoS_2_/SiN nanochannel at a bias voltage of 200 mV is recorded over 5 min [Fig. S8] for translocation events analysis as shown in Fig. 4(a) and Fig. 4(b). A representative 3-seconds segment highlighting characteristic signal features was selected for detailed analysis and presented in Fig. 4(c). Compared to previously published works of BSA translocation through single-layer MoS_2_ nanopore or of BSA-SDS translocation through few nanometers size SiN nanopore, we observed a significant enhancement of the translocation dwell time, which could stem from the noncovalent interaction between the disulfide bonds from BSA chain and the S-vacancies from the MoS_2_ surface [24].

A significant increase in the baseline ionic current was observed in the MoS_2_/SiN nanochannel after the channel was incubated by low concentration SDS/KCl solution and the high concentration SDS-modified BSA solution was introduced into the nanochannel (Fig. 4c), reaching values exceeding 50 nA compared to the smaller baseline of around 20 nA measured under similar electrolyte conditions (Fig. 2c). After introducing 10 µL unfolded SDS-BSA into the cis reservoir, we observed a pronounced enhancement in the ion current (Fig. S9) accompanied by a more clear rectification effect. A non-zero ion current was also recorded in the absence of an applied transmembrane voltage, consistent with the observation with the presence of gating-bias-induced building electrical field across the channel. These collective phenomena suggest that the adsorption of excess unfolded SDS molecules onto the hydrophobic MoS_2_ surface increases its surface charge density and amplifies the proposed mechanism of localized surface charge accumulation near the channel edge. Additionally, the SDS solution itself introduces high ionic strength (due to its Na^+^ counter-ions), further elevating the bulk conductivity within the nanochannel.

## Conclusion

In summary, this study experimentally investigated the ion transport through MoS_2_/SiN nanochannels with width ~ 100 nm and depth ~ 10 nm, demonstrated its ion current tunability through gating electrode and applications on osmotic power generation and linearized BSA translocation/detection. The ambipolar nanofluidic ion transistor behavior suggests the surface charge tunability of MoS_2_ flake. Further work can be expected to extend the single nanochannel configuration to multi-channel structures with separate gate electrodes to achieve multiplex tunability which can be used as an ionic gate. The large dwell time of linearized BSA translocation indicates the potential of utilizing the interaction between molecules and MoS_2_ surface for molecule sensing. This work may facilitate future fluidic studies along with those applications.

## Supplementary Information

Supplementary Material 1.

## Data Availability

The datasets generated and/or analyzed during the current study are available from the corresponding author on reasonable request.
