# Supplementary material for: Gated MoS2/SiN nanochannel for tunable ion transport and protein translocation
Source: J Nanobiotechnology. 2026 Jan 9;24:136. doi: 10.1186/s12951-025-03991-x (PMC12882191; doi:10.1186/s12951-025-03991-x)
Supplement: Supplementary file 1 — Supplementary Material 1. [file 12951_2025_3991_MOESM1_ESM.docx]

**SUPPORTING INFORMATION**

**Gated MoS_2_/SiN Nanochannel for Tunable Ion Transport and Protein Translocation**

Shukun Weng ^1,2*^; Ali Douaki ^1,3*^; Makusu Tsutsui ^4^, German Lanzavecchia ^1,3^, Anastasiia Sapunova ^1,2^, Lorenzo Iannetti^5^, Alberto Giacomello^5^, Roman Krahne ^1^, and Denis Garoli ^1,3*^

^1^ Optoelectronics Research Line, Instituto Italiano di Tecnologia, 16163 Genova, Italy

^2^ Department of Materials Science, University of Milano-Bicocca, Via R. Cozzi 55, I-20125 Milano, Italy

^3^ Dipartimento di Scienze e Metodi dell’Ingegneria, Universitàdegli Studi di Modena e Reggio Emilia, Via Amendola, 2, 43122 Reggio Emilia, Italy

^4^ The Institute of Scientific and Industrial Research, Osaka University, Mihogaoka 8-1, Ibaraki, Osaka, 567-0047, Japan

^5^ Dipartimento di Ingegneria Meccanica e Aerospaziale, Sapienza Università di Roma, Via Eudossiana, 18, 00184 Roma, Italy


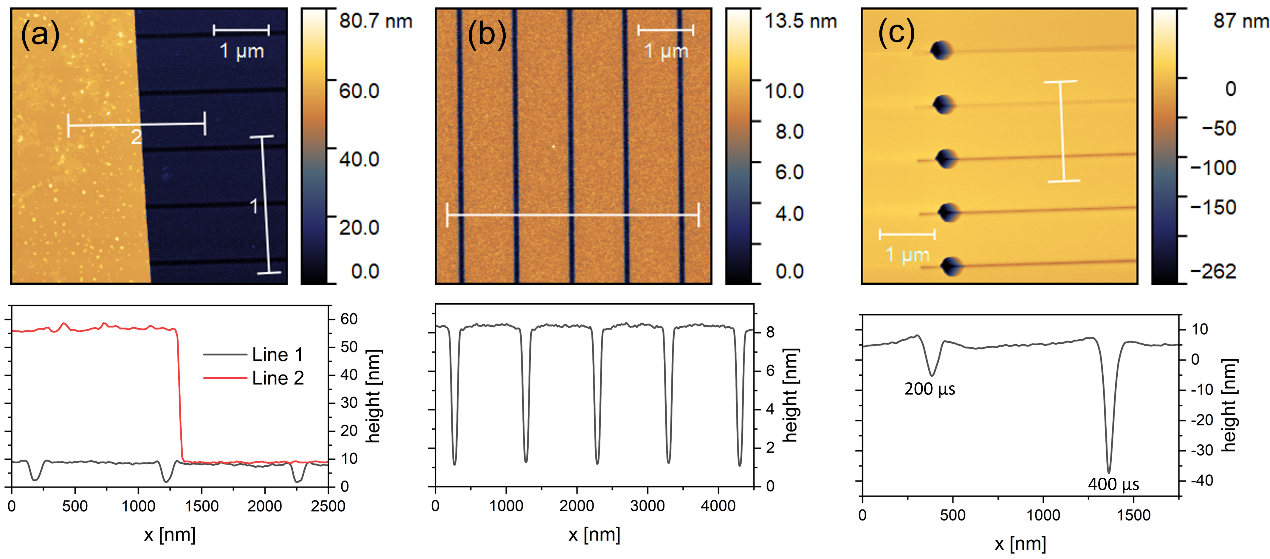


Fig. S1. AFM data of MoS_2_/SiN multichannel devices. (a) Thick MoS_2_ layers covered multichannel; (b) As fabricated multichannel by FIB with 200 μs dwell time under 18 pA; (c) Channels fabricated by FIB with different dwell time under 18 pA.


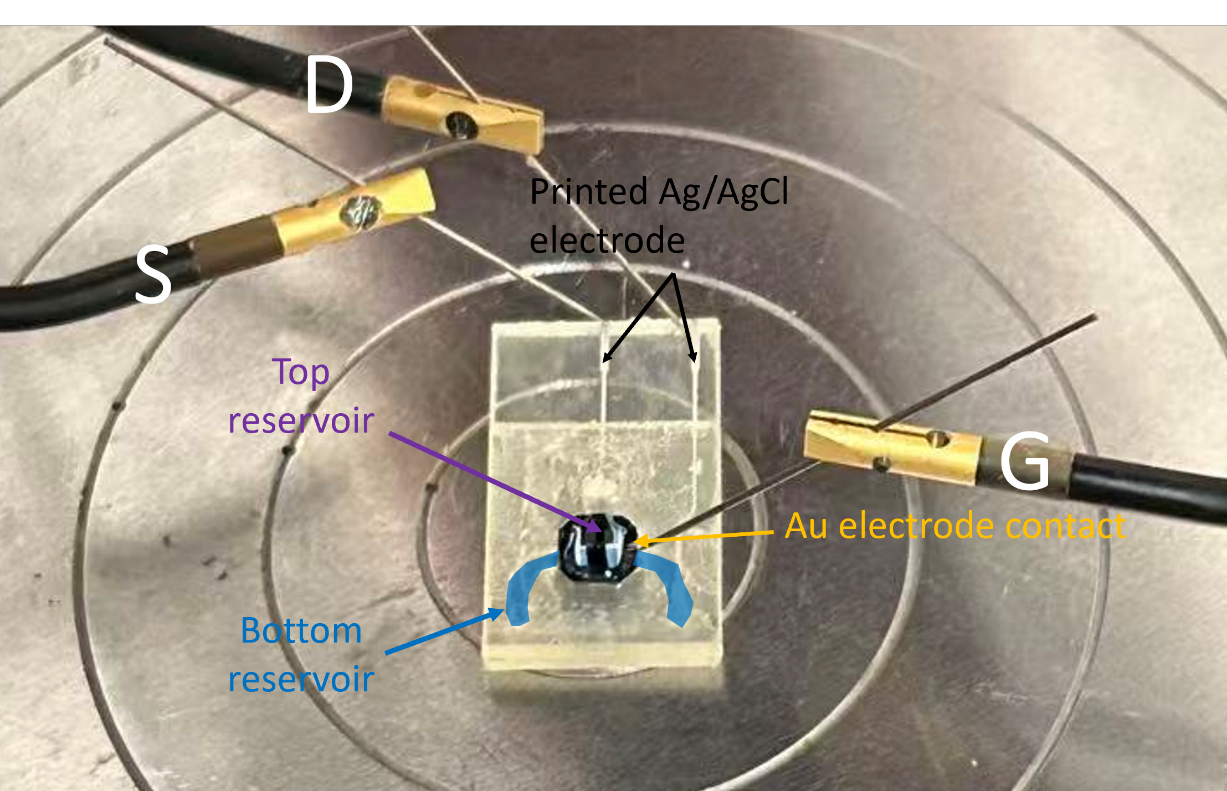


Fig. S2. Image of flow-cell used for ion gating experiment.


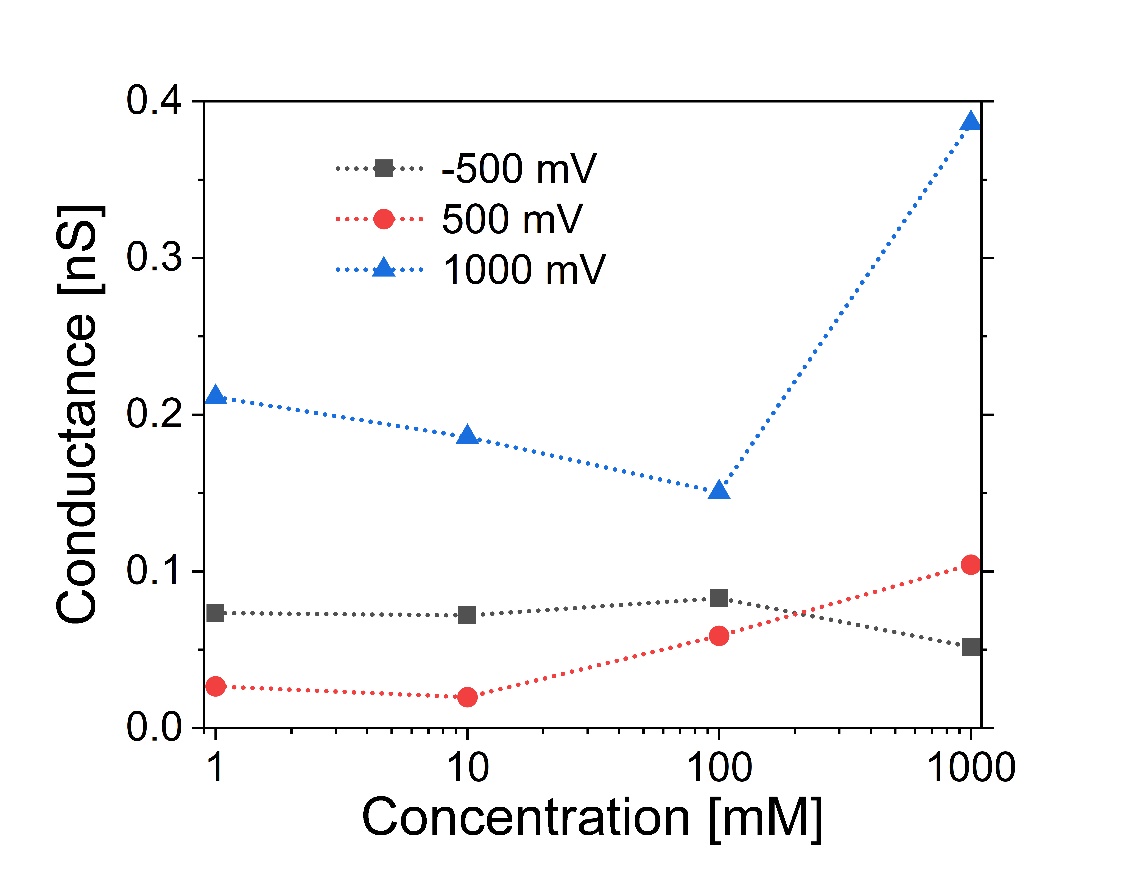


Fig. S3. The conductance of MoS_2_/SiN nanochannel within different concentrations under -500, 500 and 1000 mV potential.


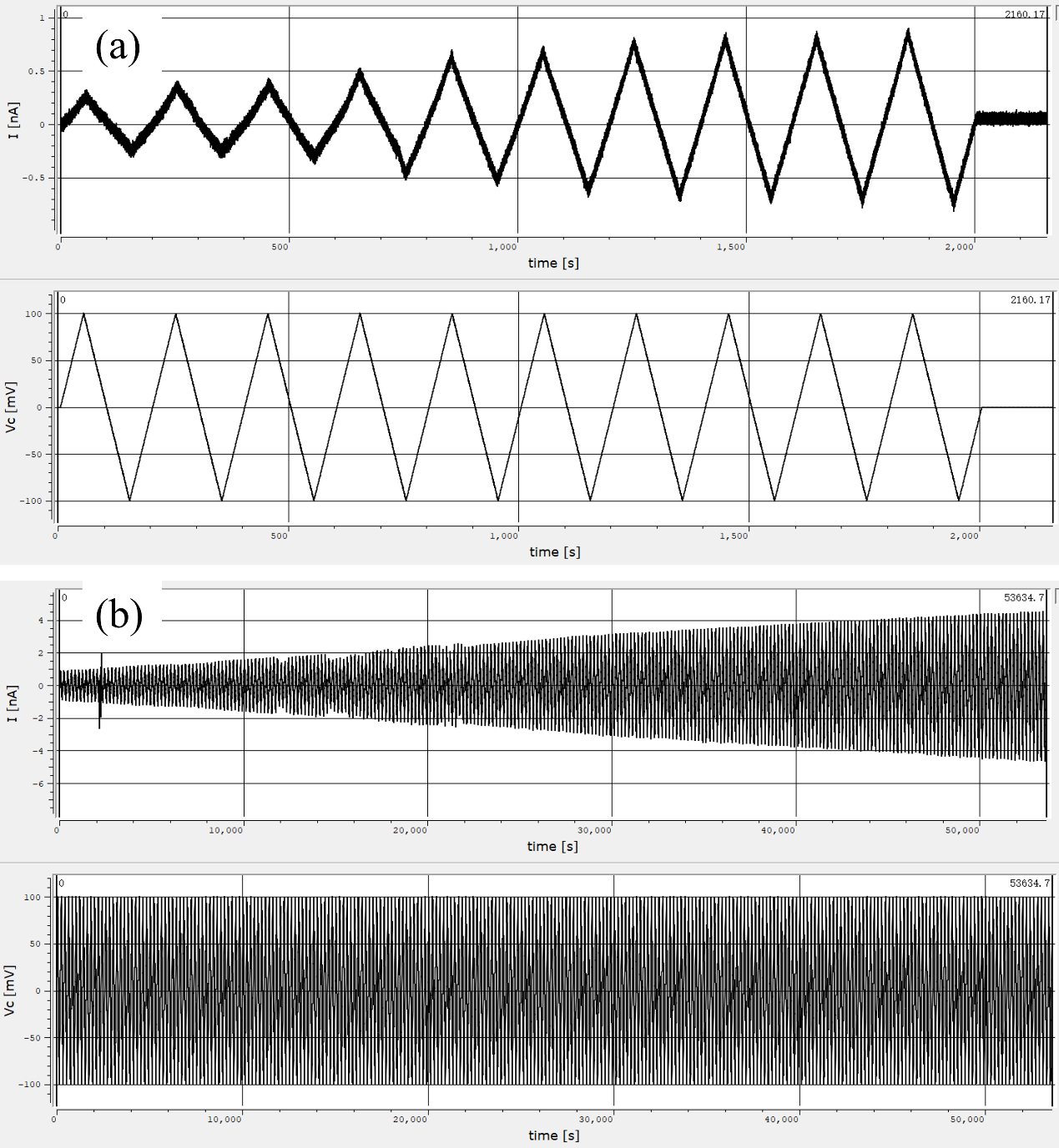


Fig. S4. Ion current traces of low voltage scanning to remove the potential contamination inside the MoS_2_/SiN channel. (a) 1^st^ to 10^th^ scan; (b) 11^th^ to 268^th^ scan.


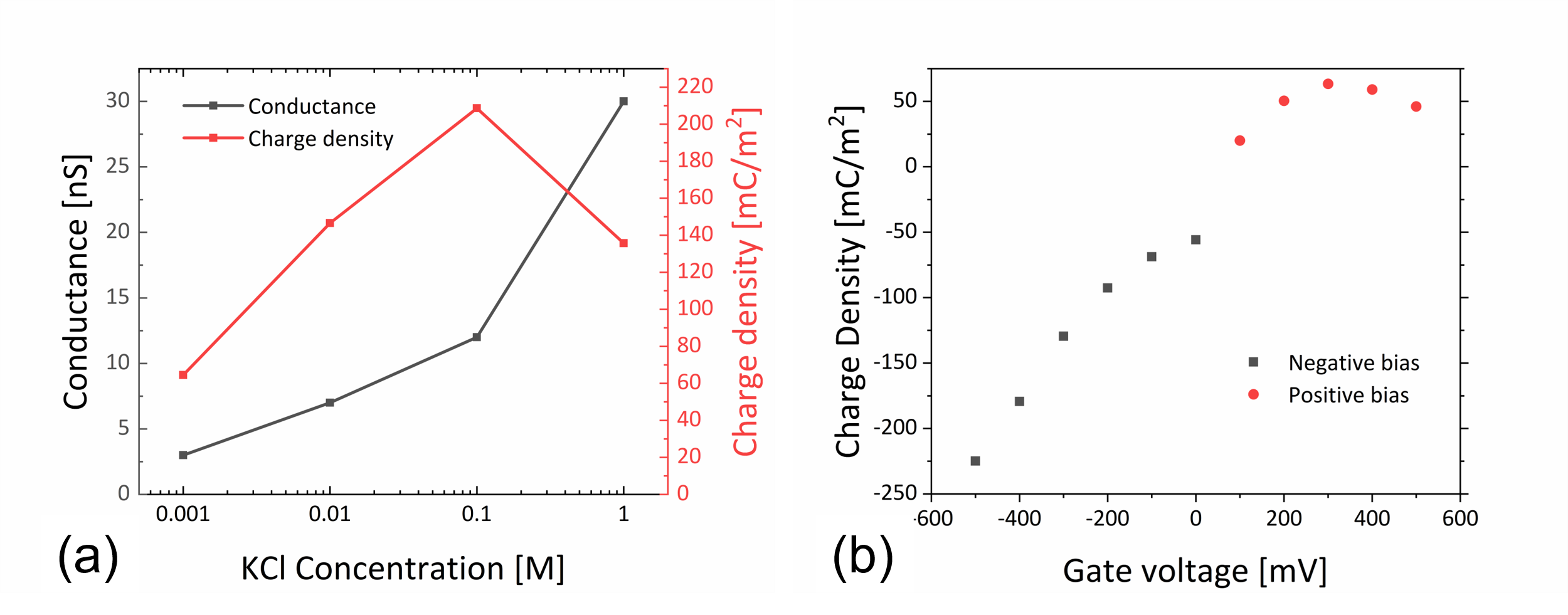


Figure S5. (a) Ionic conductance (black squares, left axis) and extracted absolute surface charge density |σ| (red circles, right axis) as a function of KCl concentration at Vg = 0 V and pH 7.5 (Device 1). (b) Gate-voltage-dependent effective surface charge density σ extracted from conductance measurements at fixed 0.01 M KCl concentration (pH 7.5) (Device 2).


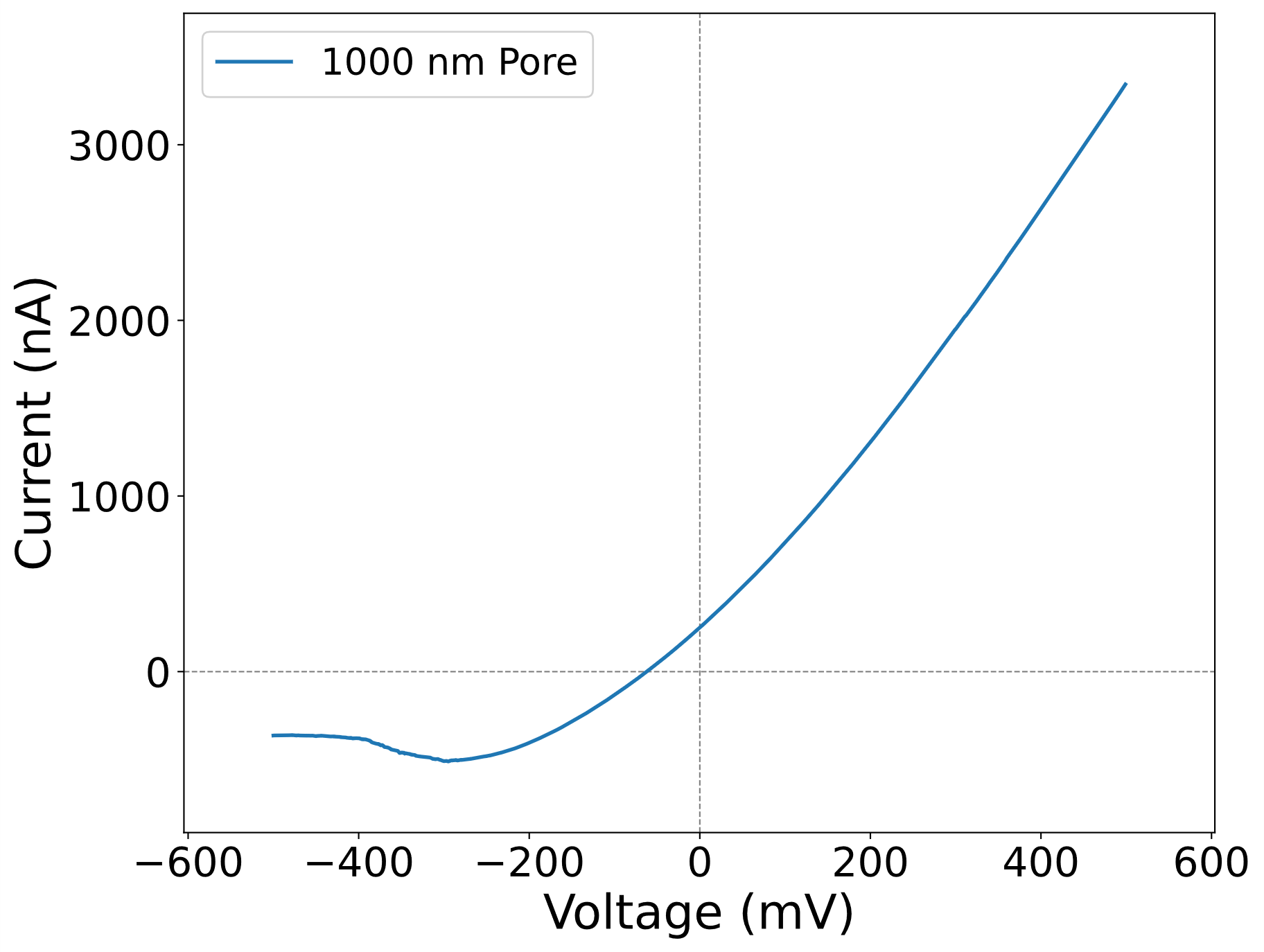


Fig. S6. IV curve of 1000 nm SiN pore under 10:1 X PBS solution gradient.


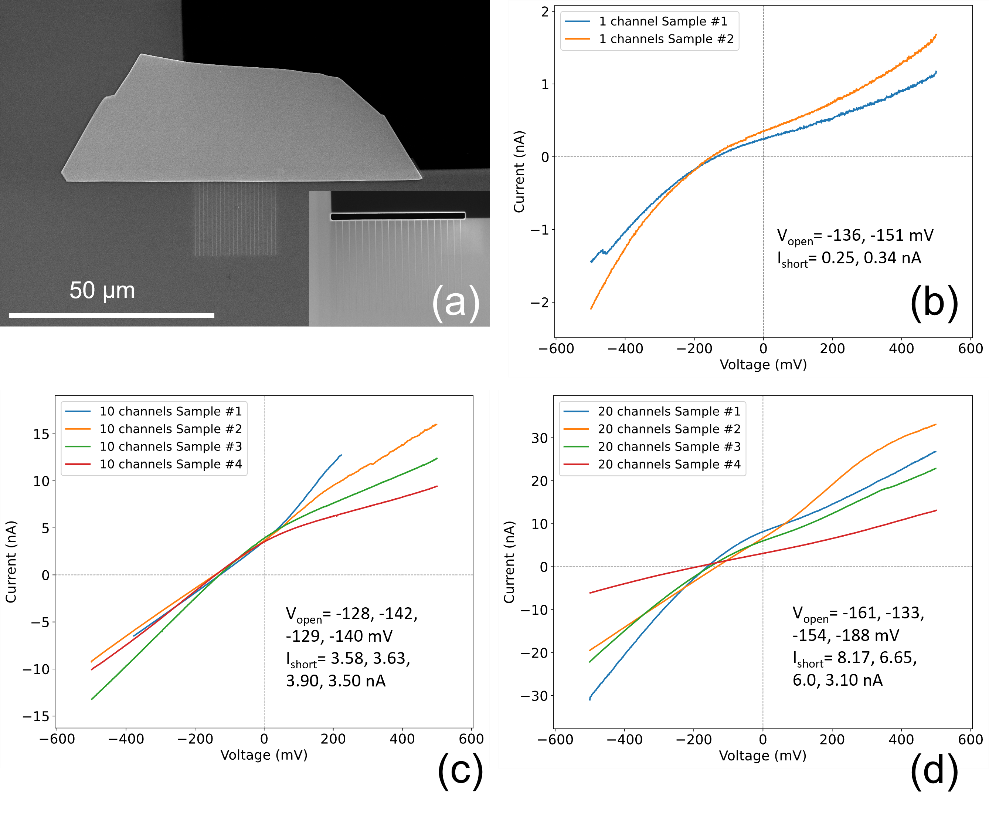


Fig. S7. Scaling of the osmotic power of MoS_2_/SiN nanochannels.


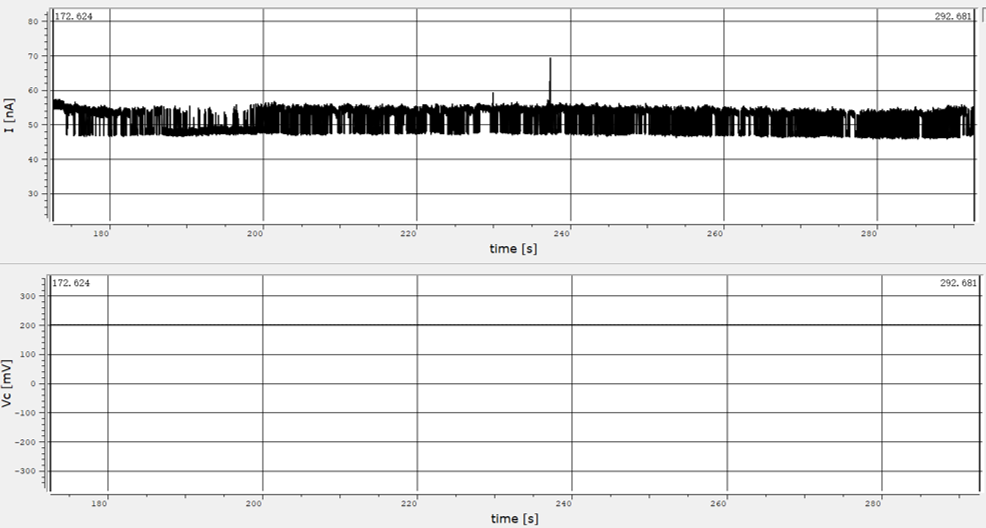


Fig. S8. BSA translocation events during 2 min recording.





Fig. S9. Ion current of 20 channel MoS_2_/SiN device before and after add 10 μL SDS-BSA solution into the reservior.

Table. S1. Comparison of osmotic power density in nanofluidic and nanopore-based energy conversion systems.

| **System** | **Geometry / Thickness** | **Electrolyte Gradient** | **Power Density (W/m²)** | **Reference** |
| --- | --- | --- | --- | --- |
| **This work: MoS₂/SiN hybrid nanochannel (single channel)** | ~10 nm depth, 100 nm width | 1X / 10X PBS | ~18,000 | This study |
| **This work: 10-channel array** | Same geometry | 1X / 10X PBS | ~14,425 | This study |
| **This work: 20-channel array** | Same geometry | 1X / 10X PBS | ~33,723 | This study |
| **PET nanochannels** | 20–40 nm | KCl gradient | 1,000–5,000 | [S1] |
| **hBN nanotube** | ~3 nm diameter | KCl gradient | ~3,000 | [S2] |
| **Single-layer MoS₂ nanopores** | ~0.7 nm | KCl gradient | 10,000–13,000 | [S3] |
| **Graphene nanopores** | 0.34 nm | NaCl/KCl gradients | 1,000–2,000 | [S4] |

References:

[S1] T. Xiao, X. Li, Z. Liu, B. Lu, and J. Zhai, The nanoscale modulation of interlayer space in two-dimensional nanoclay membranes for osmotic energy conversion, Journal of Membrane Science **695**, 122456 (2024).

[S2] A. Siria, P. Poncharal, A.-L. Biance, R. Fulcrand, X. Blase, S. T. Purcell, and L. Bocquet, Giant osmotic energy conversion measured in a single transmembrane boron nitride nanotube, Nature **494**, 455 (2013).

[S3] J. Feng, M. Graf, K. Liu, D. Ovchinnikov, D. Dumcenco, M. Heiranian, V. Nandigana, N. R. Aluru, A. Kis, and A. Radenovic, Single-layer MoS2 nanopores as nanopower generators, Nature **536**, 197 (2016).

[S4] R. C. Rollings, A. T. Kuan, and J. A. Golovchenko, Ion selectivity of graphene nanopores, Nat Commun **7**, 1 (2016).
